# Pharmacokinetics, optimal dosages and withdrawal time of amoxicillin in Nile tilapia (*Oreochromis niloticus*) reared at 25 and 30 °C

**DOI:** 10.1080/01652176.2024.2396573

**Published:** 2024-08-27

**Authors:** Tirawat Rairat, Yi-Ping Lu, Wan-Cih Ho, Hual-Jhong Ke, Chi-Chung Chou

**Affiliations:** a Department of Fishery Biology, Kasetsart University, Bangkok, Thailand; b Biology Division, Veterinary Research Institute, Ministry of Agriculture, Taipei, Taiwan; c Department of Veterinary Medicine, College of Veterinary Medicine, National Chung Hsing University, Taichung, Taiwan

**Keywords:** Antimicrobials, penicillins, fish, aquaculture, tissue residue, withdrawal period

## Abstract

Knowledge of amoxicillin (AMX) pharmacokinetics (PK) and tissue residues in fish, which is necessary for prudent drug use, remains limited. The study aimed to explore the PK characteristics of AMX in Nile tilapia (*Oreochromis niloticus*) reared at 25 and 30 °C as well as to determine optimal dosages and drug withdrawal time (WDT). In the PK investigation, the fish received a single dose of 40 mg/kg AMX *via* oral gavage, and the optimal dosage was determined by the pharmacokinetic-pharmacodynamic approach. In the tissue residue study, the fish were orally gavaged with 40 mg/kg/day AMX once daily for 5 days and the WDT was established by the linear regression analysis. The results revealed the temperature-dependent drug elimination; the clearance relative to bioavailability (CL/F) and elimination half-life at 30 °C (0.180 L/kg/h and 6.06 h, respectively) were about twice those at 25 °C (0.090 L/kg/h and 10.49 h, respectively). The optimal dosages at the minimum inhibitory concentration (MIC) of 2 μg/mL were 10.97 (25 °C) and 41.03 (30 °C) mg/kg/day, respectively. Finally, following the multiple oral administration, the muscle/skin residue of AMX on day 1 after the last dosing at 25 and 30 °C were 548 and 264 ng/g, respectively. The average tissue residues were depleted below the maximum residue limits (MRL) of 50 μg/kg on day 5 (25 °C) and 3 (30 °C), respectively, and the WDT were 6 and 4 days when rearing at 25 and 30 °C, respectively. This knowledge serves as a practical guideline for responsible use of AMX in treating bacterial diseases in Nile tilapia aquaculture.

## 1. Introduction

Amoxicillin (AMX) is a β-lactam antibiotic commonly used in both human (AphA 2014; Gilbert et al. 2022) and veterinary medicine (EMA 2008; Prescott 2013). It is also approved for aquaculture use in Taiwan (COA 2023), Thailand (Thai DoF 2020), and the European Union (EC 2023). AMX binds to penicillin-binding proteins and inhibits bacterial cell wall synthesis (AphA 2014), and mainly works against Gram-positive bacteria such as *Streptococcus* spp., *Staphylococcus* spp., and *Enterococcus* spp. (Gilbert et al. 2022). The general dosages of AMX for therapeutic use in fish range from 25 mg/kg/day twice daily for 10 days to 40–80 mg/kg/day once daily for 10 days (Reimschuessel et al. 2013). However, different regulatory authorities may have different guidelines. In Taiwan, the recommended dosage of AMX is 40 mg/kg/day (as amoxicillin trihydrate) for 3–5 days with a withdrawal time (WDT) of 5 days (COA 2023). The maximum residue limit (MRL) of 50 ng/g in the fish muscle/skin has been assigned by the European Union (EC 2023). In Thailand, other than the list of approved aquaculture drugs, neither the recommended dosage nor WDT guidelines for a specific drug is available, and the drug users are suggested to strictly follow the product labels (Thai DoF 2020).

Inland freshwater fish aquaculture in many parts of the world is dominated by Nile tilapia (*Oreochromis niloticus*) (FAO 2021, 2022). Streptococcosis, a bacterial disease mainly caused by *Streptococcus iniae* and *S. agalactiae*, is one of the most widespread diseases in Nile tilapia aquaculture (Jantrakajorn et al. 2014; Niu et al. 2020); the infected fish typically respond well when properly treating with AMX (Darwish and Ismaiel 2003; Darwish and Hobbs 2005; Lim et al. 2017). For prudent drug use, knowledge of the optimal dosage and appropriate WDT is essential. The determination of an optimal dosing regimen requires pharmacokinetic-pharmacodynamic (PK-PD) data, whereas that for WDT necessitates tissue residue study. The pharmacodynamic (PD) characteristics of AMX have been explored only in a few fishes such as olive flounder (*Paralichthys olivaceus*) (Park et al. 2016; Lim et al. 2017; Lee et al. 2023), Japanese eel (*Anguilla japonica*) (Hung et al. 2019), gilthead seabream (*Sparus aurata*) (Della Rocca et al. 2004), snubnose pompano (*Trachinotus blochii*) (Wang et al. 2015), and hybrid red tilapia (*Oreochromis mossambicus* × *O. niloticus*) (Phu et al. 2019). Likewise, only limited data regarding the WDT of AMX following multiple dosage regimens is available, which include the studies in hybrid red tilapia (Phu et al. 2019) and olive flounder (Park et al. 2016); in these studies, the WDT can range from 1 day in hybrid red tilapia to 12 days in olive flounder. For the PD parameters, the reported minimum inhibitory concentration (MIC) of AMX against sensitive strains of *Streptococcus* spp. such as *S. iniae* from Nile tilapia was 0.03–0.5 µg/mL (Lukkana et al. 2016) and that of *S. parauberis* from olive flounder was 0.03–2 µg/mL (Park et al. 2016, 2018). Sadly, AMX-resistant *S. iniae* from Asian seabass (*Lates calcarifer*) with MIC values of 32–64 µg/mL was also documented (Kumphaphat et al. 2022).

The drug’s fate in an animal body is species-dependent. The result of PK and optimal dose study obtained from one fish species may not be applicable to other fish. For example, the difference in the optimal dosages of florfenicol (FF) between Nile tilapia and Asian seabass at the same rearing temperature (25 °C) and MIC level (2 µg/mL) are large, being 2.98 and 10.9 mg/kg/day, respectively (Rairat et al. 2022, 2023a); this disparity was attributed to the differences in the absorption rate constant (Ka), elimination rate constant (K or β), and volume of distribution (Vz/F) between the two fish species, which were 1.01 1/h, 0.045 1/h, and 0.80 L/kg, respectively, for Nile tilapia (Rairat et al. 2022), and 3.29 1/h, 0.063 1/h, and 1.69 L/kg, respectively, for Asian seabass (Rairat et al. 2023a). Note that we chose FF as the reference drug for comparison because comprehensive data on its use in numerous fish species under different rearing conditions are available. This enables us to compare the PK of AMX with those of FF in the same species and similar environments/conditions (see Discussion).

Similarly, various fish species may require different WDT; for instance, following the multiple oral administration of 10 mg/kg/day FF for 5 days at 25 °C, the WDT in these two fish were 10 and 6 days, respectively (Rairat et al. 2022, 2023a) even though the WDT of FF in both cases are well within the U.S. FDA recommendation of 15 days (U.S. FDA 2023). In addition to the fish species, environmental factors especially water temperature also exert significant influences on the drug’s PK, as well as on the optimal dosage and WDT. Our previous works demonstrated that the optimal dosages of FF at 30 °C were at least twice those at 25 °C, mainly due to the faster drug elimination at the higher temperature (Rairat et al. 2022, 2023a). Unfortunately, comparable information for AMX in Nile tilapia is currently unavailable; the PK of AMX as well as its WDT in Nile tilapia has yet to be fully investigated. To the best of the authors’ knowledge, partial information on PK and WDT of AMX in tilapia can be obtained from one publication (Phu et al. 2019). In that study, the data of the peak plasma concentration (C_max_, 2.75 µg/mL), time to the peak concentration (T_max_, 1 h), area under the plasma concentration-time curve (AUC, 28.06 h·μg/mL), and terminal half-life (t_1/2_, 4.1 h) in hybrid red tilapia following AMX administration at 50 mg/kg body weight *via* medicated feed were presented. The muscle/skin AMX of all fish depleted below the MRL of 50 ng/g within 12 h after the last medication, but the WDT determination by the standard approach (i.e. linear regression analysis) was not performed (Phu et al. 2019). In the current study, the complete PK parameters, including those crucial for the optimal dosing regimen determination (namely, Ka, β, and Vz/F), of AMX in Nile tilapia were investigated at two water temperature levels. The appropriate WDT was also established using the linear regression analysis. This knowledge is necessary for the responsible use of AMX in treating bacterial infections in Nile tilapia aquaculture.

## 2. Materials and methods

### 2.1. Chemicals

Amoxicillin trihydrate reference standard was purchased from Sigma-Aldrich (St. Louis, MO, USA). Acetonitrile (HPLC grade) was purchased from Avantor Performance Materials (Center Valley, PA, USA). Potassium dihydrogen phosphate (KH_2_PO_4_) was purchased from was purchased from Fujifilm (Osaka, Japan).

### 2.2. Experimental fish

A total of 90 healthy Nile tilapia (10 fish for the PK study and 80 fish for the tissue residue study), 350–500 g body weight, were obtained from a commercial fish farm in Tainan County, Taiwan, and were kept in an outdoor concrete pond at the College of Veterinary Medicine, National Chung Hsing University, Taiwan. Each individual fish was acclimatized in a 240 L-glass aquarium tank containing freshwater at 25 °C and 30 °C for 6–7 days before drug administration. The water temperature was controlled by 500 W-aquarium heaters (Mr. Aqua, Taiwan) in an air-conditioned room (for 25 °C). Dissolved oxygen (DO) was maintained at ≥ 5.0 mg/L and pH was in the range of 7.5–8.0. Temperature, DO, and pH were measured by a portable water quality meter (Lutron WA-2017SD, Lutron Electronics, Coopersburg, PA, USA). The animal study was approved by the Institutional Animal Care and Use Committee of National Chung Hsing University (IACUC approval No: 110-093).

### 2.3. Pharmacokinetic study

#### 2.3.1. Drug administration and blood collection

Five Nile tilapia at each temperature level were orally administered with AMX solution *via* an 8.4 cm-stainless steel oral gavage tube. The AMX suspension was prepared by mixing AMX standard powder with double distilled water (DDW) to attain the final concentration of 40 mg/mL (freshly prepared before the drug administration). Then, it was administered at the rate of 1 mL/kg body weight to attain the desired dose of 40 mg/kg. Blood samples (0.4–0.5 mL) from each individual Nile tilapia were serially collected from the caudal vessel using a 1 mL-syringe with 22 G needle without anticoagulant at predetermined time points: 0.25, 0.5, 1, 2, 4, 8, 12, 24, and 36 h post-administration. The blood samples were allowed to clot at room temperature and then centrifuged at 2000 × g for 10 min. The supernatants (serum) were collected and kept at −20 °C until analysis. During the blood sampling, no anesthetic agent was used to avoid any potential interactions between the anesthetic and the antibiotic that could interfere with the results (Rairat et al. 2021).

#### 2.3.2. Serum sample preparation and HPLC analysis

The protocols of serum sample preparation and HPLC analysis of AMX followed Park et al. (2016). One mL of acetonitrile was added per 0.2 mL serum sample in a 2 mL Eppendorf microtube and was vortex-mixed. Then, they were subjected to ultrasonication for 10 min. After centrifugation at 12,000 × g for 10 min, the supernatant was transferred to a 5 mL tube. The supernatant was dried using a sample concentrator (SP Genevac miVac Duo Concentrator, Suffolk, England), and the residue was reconstituted with 200 μL mobile phase (see below) and then filtered through 0.2-μm nylon syringe filter prior to the HPLC analysis. The mobile phase consisted of 50 mM KH_2_PO_4_ buffer (pH 3) and acetonitrile (95:5). The HPLC system consisted of HPLC pump, (1260 Infinity II, Agilent Technologies, Santa Clara, CA, USA), UV detector (G7115A, Agilent Technologies, Waldbronn, Germany), vial sampler (G7129A, Agilent Technologies, Waldbronn, Germany), and C-18 column with 5 µm particle size, 150 × 4.6 mm (Apollo, Hichrom, UK). The injection volume and the flow rate were 50 μL and 1 mL/min, respectively. The UV detection wavelength was set at 229 nm.

To establish the matrix-matched calibration curves for quantification of AMX concentration in the Nile tilapia serum, the AMX standards were spiked into the blank serum to attain the final concentrations of 0.04, 0.1, 0.5, 1, 10, 25, 50 μg/mL. Then, they were extracted and analyzed by the HPLC method described above. The matrix calibration curve of the serum AMX was linear over the range of 0.04–50 μg/g with the weighted r^2^ of 0.9987, the limit of detection (LOD) was 4 ng/mL, and the limit of quantification (LOQ) was 13 ng/mL. The LOD and LOQ were calculated by 3.3*σ/S and 10*σ/S, respectively (σ = standard deviation of the y-intercept of the regression line; S = slope of the calibration curve). The extraction recovery was 83–112%. The precision was < 8.7%, and the accuracy was > 96%.

#### 2.3.3. Pharmacokinetic analysis and optimal dosing regimen determination

The PK parameters were determined by the 2-compartmental model with a weighting scheme of 1/C by PKSolver 2.0 software (China Pharmaceutical University, Nanjing, China) (Zhang et al. 2010). The PK parameters were the absorption rate constant (Ka), absorption half-life (t_1/2 Ka_), distribution rate constant (α), distribution half-life (t_1/2α_), elimination rate constant (β), elimination half-life (t_1/2β_), transfer rate constant from the central (1) to peripheral (2) compartment (k_12_), transfer rate constant from the peripheral (2) to central (1) compartment (k_21_), elimination rate constant from the central compartment (k_10_), maximum serum concentration (C_max_), time to reach C_max_ (T_max_), area under the serum concentration-time curve (AUC), volume of distribution (Vd) of the central compartment relative to bioavailability (Vc/F), Vd during the elimination phase relative to bioavailability (Vz/F), Vd at steady-state relative to bioavailability (Vss/F), clearance relative to bioavailability (CL/F), and mean residence time (MRT). The pharmacokinetic-pharmacodynamic (PK-PD) equation for a time-dependent antimicrobial drug, which is based on the Ka, β, and Vz/F (PK parameters), and minimum inhibitory concentration (MIC) (PD parameter) against target bacteria, was adopted to determine the optimal dosing regimens as previously described (Rairat et al. 2019a).

### 2.4. Tissue residue study

#### 2.4.1. Drug administration and tissue collection

At each temperature level, 40 Nile tilapia were orally administered with AMX solution *via* oral gavage at the dose of 40 mg/kg/day once daily for 5 consecutive days. On days 1,3, 5, and 7 after the last dose, the fish (*n* = 10/time point) were humanely sacrificed by rapid severance of the head and brain from the spinal cord, followed by pithing of the brain according to the AVMA Guidelines (AVMA 2020) and the muscle/skin (muscle plus skin in natural proportion) was collected. All tissue samples were stored at −20 °C until analysis.

#### 2.4.2. Tissue sample preparation and HPLC analysis

The protocols of tissue sample preparation and HPLC analysis of AMX followed Park et al. (2016). An amount of 5 g of the homogenized muscle/skin (in its natural proportion) sample was accurately weighed into a 50 mL tube; the extraction solution of 20 mL 0.1 M KH_2_PO_4_ buffer (pH 2.5) was added with vortex mixing for 5 min. The mixture was centrifuged at 3800 × g for 20 min. The upper solution (about 15 mL) was transferred to a clean tube, and was subjected to a solid phase extraction (SPE) process. A 500 mg (6 mL) Bone Elut C18 SPE cartridge (Agilent, USA) was first activated with 5 mL methanol followed by 5 mL DDW. The supernatant was loaded onto the cartridge. Then washed with 5 mL DDW. Elution was then carried out with 5 mL DDW:ACN (60:40) and combined into a 5-mL tube and evaporated to dryness at 45 °C using a sample concentrator. The residue was reconstituted with 500 μL DDW and then filtered through a 0.2 μm-nylon syringe filter prior to the HPLC analysis. The mobile phase consisted of 50 mM KH_2_PO_4_ buffer (pH 3) and acetonitrile (92:8). The injection volume and the flow rate were 50 μL and 1 mL/min, respectively. The UV detection wavelength was set at 229 nm.

To establish the matrix-matched calibration curves for quantification of AMX concentrations in Nile tilapia muscle/skin, the AMX standard was spiked into the blank muscle/skin to attain the final concentrations of 0.05, 0.1, 0.5, and 1 μg/g. Then, they were extracted and analyzed by the HPLC method described above. The matrix-matched calibration curve of the muscle/skin AMX was linear over the range 0.05–1 μg/g with the weighted r^2^ of 0.9986, the LOD was 9 ng/g, and the LOQ was 28 ng/g. The extraction recovery was 61–83%. The precision was < 2%, and the accuracy was > 97%.

#### 2.4.3. Determination of withdrawal time

The withdrawal time (WDT) of AMX in the Nile tilapia muscle/skin at each temperature was determined by the linear regression analysis using WT 1.4 software (EMA 2008). The WDT is the time when the upper one-sided 95% tolerance limit with a 95% confidence is below the MRL of 50 ng/g (EC 2023). In addition, the depletion half-life (t_1/2_) of AMX in the muscle/skin was calculated by PKSolver 2.0.

### 2.5. Statistical analysis

The differences in PK parameters between the two temperatures were analyzed by independent t-test using IBM SPSS Statistics version 27 software (IBM Corporation, Armonk, NY, USA). The *p*-value of < 0.05 was considered statistically significant.

## 3. Results

**Figure 1.**
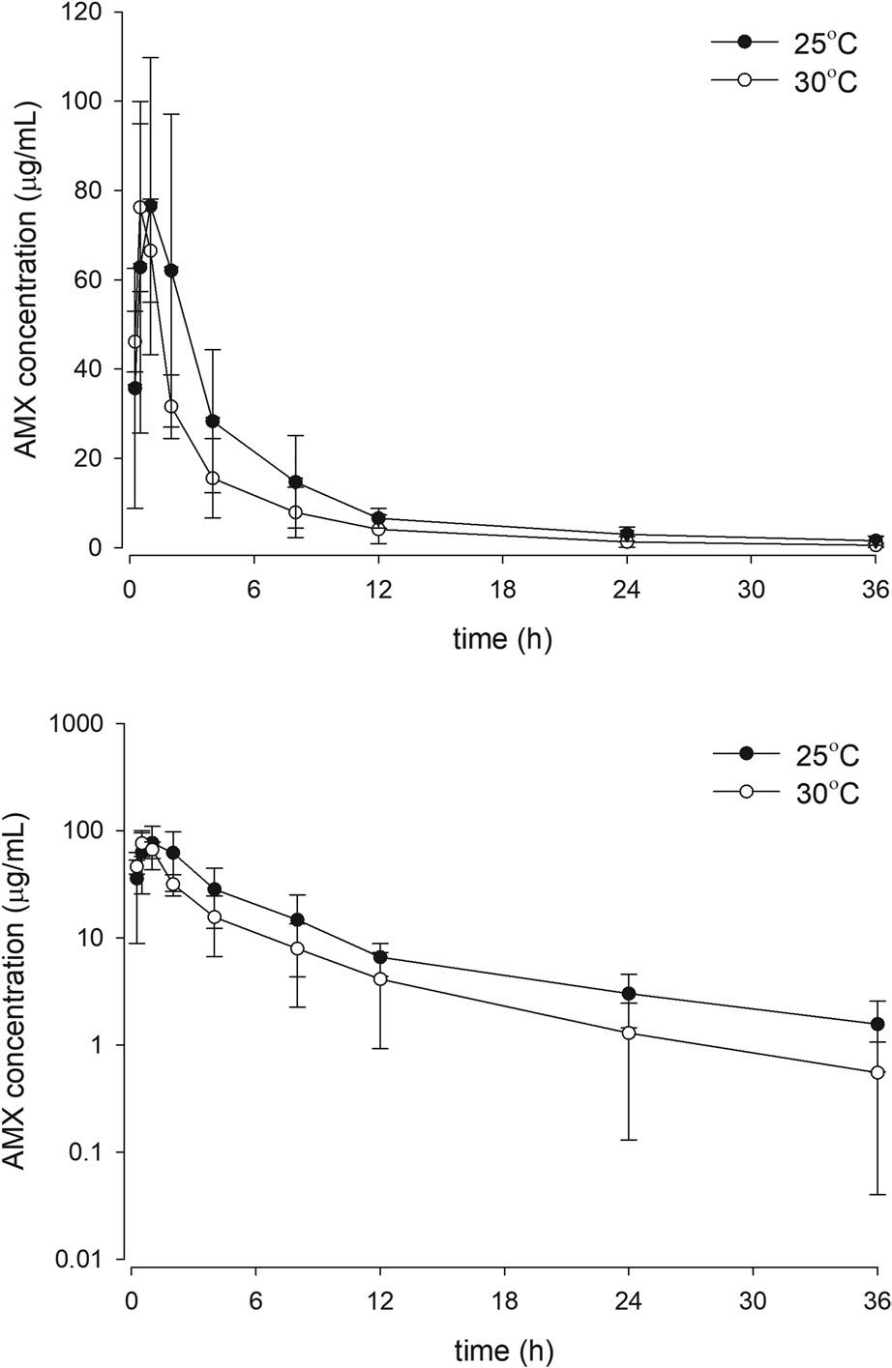
Linear (above) and semi-logarithmic plots (below) of serum concentration-time profile (mean ± SD) of amoxicillin in Nile tilapia following single oral administration of 40 mg/kg amoxicillin trihydrate at 25 and 30 °C (*n* = 5).

The PK parameters of AMX at the two temperatures are given in Table 1 and their serum concentration-time profiles are shown in Figure 1. At 25 °C, the t_1/2 Ka_ and t_1/2α_ of AMX were 0.52 and 0.94 h, respectively. The serum concentration reached T_max_ (76.04 µg/mL) at 1.23 h. Then it was eliminated with the t_1/2β_ of 10.49 h. The AUC, Vz/F, CL/F, and MRT were 469.81 h·µg/mL, 1.46 L/kg, 0.090 L/kg/h, and 9.67 h, respectively. At 30 °C, the t_1/2 Ka_ (0.33 h) and C_max_ (70.44 µg/mL) were lower than at 25 °C, but the differences were not statistically significant. The three Vd parameters (namely, Vz/F, Vc/F, and Vss/F) were also not affected by the changing temperature. However, the fish at higher water temperature eliminated the drug faster, as indicated by the doubled CL/F at 30 °C (0.180 L/kg/h) in comparison with 25 °C. Likewise, the t_1/2β_ (6.06 h) and MRT (5.45 h) were also altered by the warmer temperature to a similar extent.

**Table 1.** Pharmacokinetic parameters (mean ± SD) of 40 mg/kg amoxicillin trihydrate in Nile tilapia following PO administration at 25 and 30 °C (*n* = 5).

PK parameters	25 °C	30 °C
Ka (1/h)	1.32 ± 0.55^a^	2.07 ± 0.87^a^
t_1/2 Ka_ (h)	0.52 ± 0.25^a^	0.33 ± 0.12^a^
α (1/h)	0.74 ± 0.33^a^	1.48 ± 0.40^b^
t_1/2α_ (h)	0.94 ± 0.69^a^	0.47 ± 0.14^b^
β (1/h)	0.066 ± 0.017^a^	0.114 ± 0.009^b^
t_1/2β_ (h)	10.49 ± 3.22^a^	6.06 ± 0.49^b^
k_12_ (1/h)	0.31 ± 0.24^a^	0.63 ± 0.43^a^
k_21_ (1/h)	0.14 ± 0.06^a^	0.27 ± 0.11^b^
k_10_ (1/h)	0.36 ± 0.09^a^	0.69 ± 0.19^b^
C_max_ (µg/mL)	76.04 ± 39.43^a^	70.44 ± 7.99^a^
T_max_ (h)	1.23 ± 0.54^a^	0.65 ± 0.12^b^
AUC (h·µg/mL)	469.81 ± 123.65^a^	246.32 ± 83.43^b^
Vz/F (L/kg)	1.46 ± 0.45^a^	1.35 ± 0.31^a^
Vc/F (L/kg)	0.25 ± 0.06^a^	0.26 ± 0.08^a^
Vss/F (L/kg)	0.78 ± 0.45^a^	0.78 ± 0.16^a^
CL/F (L/kg/h)	0.090 ± 0.023^a^	0.180 ± 0.067^b^
MRT (h)	9.67 ± 3.01^a^	5.45 ± 2.22^b^

The means of half-lives are harmonic mean whereas the means of the other PK parameters are arithmetic mean. Means with different superscripts in each row are significantly different from each other (*p* < 0.05).

The changes in PK parameters due to the temperature effect resulted in temperature-dependent optimal doses. The determined optimal doses of AMX are shown in Table 2. At any MIC level, the optimal dosage at 30 °C was more than triple compared to that of 25 °C. For instance, at the MIC of 2 µg/mL, the optimal doses at 25 and 30 °C were 10.97 and 41.03 mg/kg/day, respectively.

**Table 2.** Determined optimal dosing regimens (mg/kg/day) of amoxicillin trihydrate in Nile tilapia (*n* = 5) at 25 and 30 °C and different MIC values.

	25 °C	30 °C
MIC = 1 µg/mL	5.48 ± 2.18 (2.61–8.36)	20.52 ± 7.11 (11.25–28.44)
MIC = 2 µg/mL	10.97 ± 4.36 (5.23–16.72)	41.03 ± 14.34 (22.50–56.88)
MIC = 3 µg/mL	16.45 ± 6.54 (7.84–25.08)	61.55 ± 21.34 (55.76–85.31)
MIC = 4 µg/mL	21.94 ± 8.72 (10.46–33.44)	82.07 ± 28.46 (74.35–113.75)

Note. The optimal dosing regimens were presented as mean ± SD. The values in parenthesis indicated the minimum and maximum range of the determined dosage.

**Figure 2.**
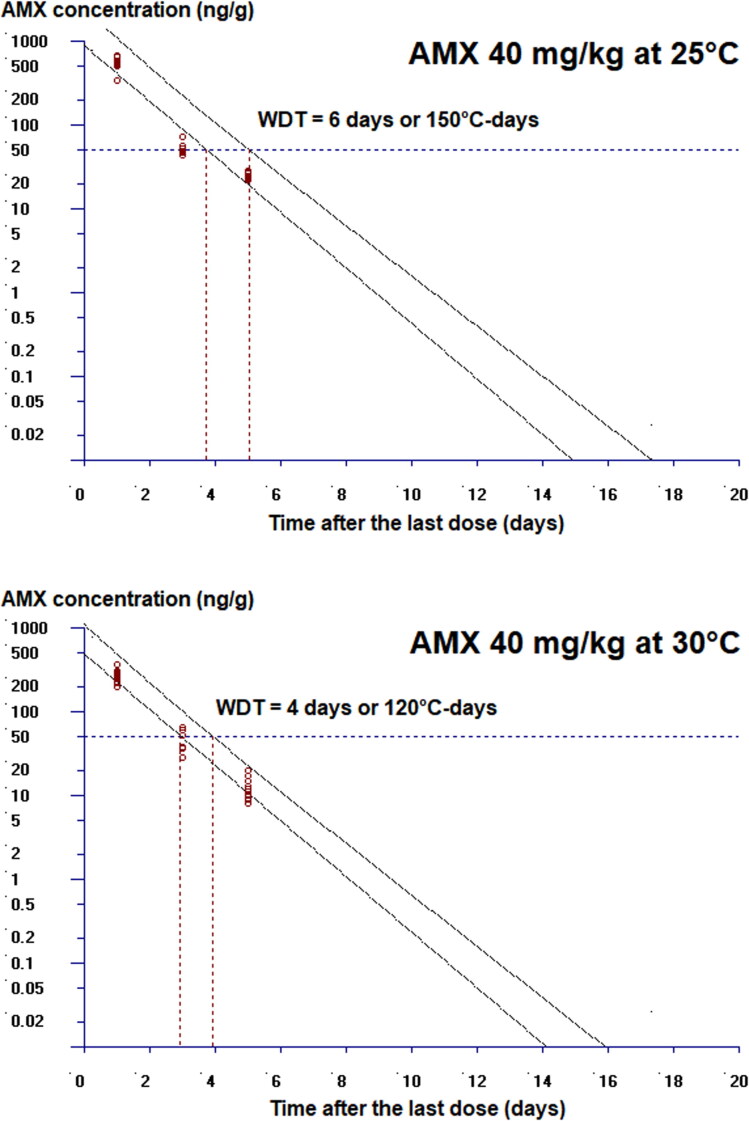
Tissue residue depletion of amoxicillin in the muscle/skin of Nile tilapia following multiple oral administration of 40 mg/kg amoxicillin trihydrate once daily for 5 days at 25 and 30 °C (*n* = 10). The lower line represents the linear regression line, while the upper line represents its 95% upper tolerance limits with 95% confidence.

The influence of water temperature on tissue residue and drug WDT was also evident (Table 3). Following the oral administration of 40 mg/kg AMX once daily for 5 days, the average AMX concentrations in the muscle/skin of Nile tilapia reared at 25 °C were always higher than at those at 30 °C. On day 1 after the last dose, the muscle/skin concentration of the 25 °C group was 547.51 ng/g, whereas those reared at 30 °C contained only half of this level (i.e. 264.15 ng/g). On day 5, AMX residues were depleted to 25.60 and 12.70 ng/g, respectively, but AMX was no longer detected on day 7 for both groups. As a result, the WDT at 25 °C (6 days or 150 °C-days) was longer than at 30 °C (4 days or 120 °C-days) (Figure 2). Nevertheless, when the t_1/2_ of AMX in the muscle/skin was calculated, it turned out that the t_1/2_ at the two temperatures were comparable, namely 21.73 and 21.93 h at 25 and 30 °C, respectively (Table 3).

**Table 3.** Amoxicillin concentration (ng/g) in the muscle/skin of Nile tilapia following multiple oral administration of 40 mg/kg amoxicillin trihydrate once daily for 5 days at 25 and 30 °C (*n* = 10) and their half-lives (t_1/2_).

Time after the last dose	25 °C	30 °C
Day 1	547.51 ± 91.46	264.15 ± 49.22
Day 3	54.21 ± 10.50	40.59 ± 14.34
Day 5	25.60 ± 2.07	12.70 ± 3.71
Day 7	ND	ND
t_1/2_ (h)	21.73	21.93

ND, not detected.

## 4. Discussion

Our understanding of AMX PK in fishes remains limited, with comprehensive data currently available for only two species: olive flounder (Park et al. 2016; Lim et al. 2017; Lee et al. 2023) and Japanese eel (Hung et al. 2019). For other fish species including tilapia, only partial PK information can be found in the literature. Phu et al. (2019) reported a much lower plasma C_max_ (2.75 μg/mL) and AUC (28.06 h·μg/mL) of AMX in hybrid red tilapia at 28–30 °C compared to our results at a comparable temperature level (30 °C), which were 70.44 μg/mL and 246.32 h·μg/mL, respectively. The disparity of the C_max_ between the two studies can be attributed to the difference in drug administration methods. In the present work, oral gavage administration led to rapid drug absorption, resulting in a high peak concentration. Conversely, in the study by Phu et al. (2019), where medicated feed was used, additional time was required for the drug molecules to be released from the surface of pelleted feed. This resulted in slower drug absorption and consequently lower peak concentration (Rairat et al. 2023b). Furthermore, the consistently low levels of AMX in the case of hybrid red tilapia also arise from a problem associated with the medicated feed preparation in which the analyzed concentrations of AMX in the feed were only about 12–45% of the intended levels (Phu et al. 2019). Unsurprisingly, AMX residue in the muscle/skin of the hybrid red tilapia was also considerably lower than that of Nile tilapia in our study; for instance, AMX concentration in the hybrid red tilapia’s muscle/skin at 12 and 24 h after the last dose were 5.6 ng/g and < LOQ, respectively (Phu et al. 2019), whereas the residue in Nile tilapia at 24 h after the last dose was 264 ng/g.

AMX has a very short serum/plasma t_1/2_ compared to other antibacterial drugs. The reported AMX t_1/2_ in olive flounder at 22–23 °C following a single intramuscular (IM) injection was about 10 h (Park et al. 2016; Lim et al. 2017; Lee et al. 2023) and that of Japanese eel at 25 °C after a single oral administration was 5.93 h (Hung et al. 2019). By comparison, those of FF in olive flounder and Japanese eel at comparable temperatures and using the same administration route were 43 h (Lim et al. 2011) and 15 h (Lin et al. 2015), respectively. In this study, the t_1/2_ of AMX in Nile tilapia maintained at 30 °C was 6.06 h, comparable to the reported AMX t_1/2_ in hybrid red tilapia (i.e. 4.1 h at 28–30 °C) (Phu et al. 2019), but shorter than FF t_1/2_ in Nile tilapia reared under similar experimental conditions which was 8.93 h (Rairat et al. 2022). This observation can be attributed to the physicochemical properties of the drug molecules, particularly the hydrophilic nature of AMX and other penicillins antibiotics that are characterized by limited tissue distribution (small Vd) and short t_1/2_ (Prescott 2013; Trevor et al. 2015; Gilbert et al. 2022). On the contrary, FF and other amphenicol antibiotics such as thiamphenicol are more lipophilic, thereby can be well-distributed into tissues and have longer t_1/2_ (Dowling 2013). Since only drug molecules in the serum/plasma can reach the organs responsible for drug elimination (such as the kidney, liver, and gill), a higher Vd contributes to a longer t_1/2_ and *vice versa* (Toutain and Bousquet-Mélou 2004).

Given that AMX is a polar compound, it might be expected that AMX would have a relatively small Vd. However, the Vss/F of AMX in Nile tilapia is 0.78 L/kg (Table 1), exceeding that of the more lipophilic drug FF which is 0.67–0.71 L/kg (Rairat et al. 2022). The most plausible explanation lies in the difference in oral bioavailability. Our previous investigations discovered that the oral bioavailability of FF in Nile tilapia is typically about 80–90% (Rairat et al. 2019b, 2020). Unfortunately, comparable data is lacking for AMX in Nile tilapia. According to one study, the oral bioavailability of AMX in gilthead seabream is only about 0.3% (Della Rocca et al. 2004). If a similar extent of bioavailability exists in Nile tilapia, the absolute Vd of AMX would be significantly decreased, thereby in line with the initial expectation. The fact that the AMX residue in the muscle/skin was much lower than that of FF, despite a four-fold increase in dosage, also supports the presumption of low oral bioavailability and relatively small absolute Vd of AMX in Nile tilapia; for instance, on day 1 of drug withdrawal, the drug concentrations in the Nile tilapia’s muscle/skin following the multiple oral administrations of AMX (40 mg/kg) and FF (10 mg/kg) at 25 °C were 0.55 and 8.35 µg/g, respectively (Table 3 and Rairat et al. 2022). Nevertheless, to ascertain the exact values of oral bioavailability and absolute Vd of AMX in Nile tilapia, a future study using an intravenous (IV) route of administration is necessary.

The PK parameters obtained from the PK study were used to calculate the optimal dosing regimen at different MIC and temperature levels. The results indicate that the dose recommended by Taiwan’s Council of Agriculture (i.e. 40 mg/kg/day) (COA 2023) was high enough to treat susceptible bacteria at 25 °C, but may not be effective against pathogenic bacteria with the MIC value higher than 2 µg/mL at 30 °C. On the same note, when used for fish reared at lower temperatures or when targeting lower MIC values, the currently regulated dosages might be more than necessary. When the optimal dosage of AMX was compared with that of FF in Nile tilapia reared at similar conditions, it appeared that the calculated optimal dosage of AMX was about 4–5 times higher than that of FF. The optimal dosing regimens of AMX and FF in Nile tilapia at 30 °C and MIC of 2 µg/mL were 41.03 and 8.40 mg/kg/day, respectively (Rairat et al. 2022). This dosage ratio is similar to the recommended dosage of AMX (40 mg/kg) and FF (10 mg/kg) in Nile tilapia. The dosage difference could be due to the faster drug elimination (β) and more extensive drug distribution relative to bioavailability (Vz/F) of AMX compared to FF (Rairat et al. 2022).

Most studies that investigated the effect of water temperature on the rate of drug elimination in the muscle (or muscle/skin) of fish usually reported faster drug depletion at a higher temperature. This includes several antimicrobial agents such as florfenicol (Huang et al. 2019; Yang et al. 2020; Rairat et al. 2022, 2023a), enrofloxacin (Xu et al. 2023), sarafloxacin (Tyrpenou et al. 2003), oxolinic acid (Rigos et al. 2002; Samuelsen 2006; Romero González et al. 2010), flumequine (Romero González et al. 2010), oxytetracycline (Romero González et al. 2010), and sulfadiazine (Romero González et al. 2010; Xu et al. 2022). Although the muscle is not considered a drug-eliminating organ, the increased cardiac output and blood circulation at the warmer temperature (Barron et al. 1987) potentially speed up drug transport from muscle to elimination sites such as gill, kidney, and liver (Matthee et al. 2023), thereby shortening the muscle t_1/2_ and drug resident time. The fact that the k_21_, the transfer rate constant from the peripheral compartment (including muscle tissue) to the central compartment (including serum), was often increased with the increasing temperature (Table 1 and Rairat et al. 2022) is also in agreement with this notion. However, this was not the case in the present study in which the AMX elimination rate from the Nile tilapia’s muscle/skin appears independent of water temperature; the AMX t_1/2_ at both temperature levels were about 22 h. The true reason why the muscle/skin depletion t_1/2_ was insensitive to temperature change is unknown. Further research is needed to understand why water temperature does not affect the drug depletion rate in certain circumstances.

In any fish species, it is widely acknowledged that the numerical value of each PK parameter is unique for a specific drug under distinct environmental conditions. However, the magnitude of PK alterations due to temperature effect appears similar across various drug classes. In Nile tilapia, an increment of 5 °C water temperature (from 25 to 30 °C) led to approximately two-fold differences in the α, β, k_12_, k_21_, k_10_, T_max_, and CL/F of AMX, whereas the C_max_ and Vd/F remained unaffected. Interestingly, comparable changes in these PK parameters were also observed for FF in Nile tilapia (Rairat et al. 2022). Likewise, a temperature increase from 5 to 10 °C produced about a 1.5-fold increase in the β of both oxolinic acid (from 0.0048 to 0.0071 1/h) (Björklund et al. 1992) and oxytetracycline (from 0.0033 to 0.0048 1/h) in rainbow trout (*Oncorhynchus mykiss*) (Björklund and Bylund 1990). This consistent pattern suggests that the extent of temperature-induced PK alterations might be independent of (or less determined by) drug type, and primarily stems from changes in certain physiological processes such as increased cardiac output and blood flow (Barron et al. 1987), elevated metabolic rate (Clarke and Johnston 1999; Killen et al. 2010), and enhanced enzyme activities at a higher temperature (Buckman et al. 2007; Amutha and Subramanian 2010). In addition, the extent to which drug is eliminated by other possible drug eliminating organs like gill and kidney may also be a confounding factor. The implication is that if we were to study the temperature effect on a new drug in Nile tilapia cultured at 25 and 30 °C, a similar magnitude of PK changes (namely, about two-fold differences in the α, β, k_12_, k_21_, k_10_, T_max_, and CL/F) would likely be seen. However, whether this conclusion holds true across various drug types requires further investigation.

The studies of WDT of AMX in fishes, particularly those employing the standard linear regression analysis method, are rare. To the author’s knowledge, there are only two publications that use this approach. After a single IM injection of 40 mg/kg and a 7-day multiple IM injection of 37.5 mg/kg AMX in olive flounder at 22–23 °C, the WDT were determined as 8 and 12 days, respectively (Park et al. 2016; Lim et al. 2017). Other studies merely reported the time when the average drug residue depleted below the MRL of 50 ng/g; they were 7 days after a single IM injection of 40 mg/kg AMX in olive flounder at 22 °C (Lee et al. 2023), 3 days after a single oral dosing of 40–80 mg/kg AMX in Japanese eel at 25 °C (Hung et al. 2019), 1–2 days after a 5-day multiple oral administration of 40 mg/kg AMX in snubnose pompano at 25–27 °C (Wang et al. 2015), and 1 day after a 10-day multiple oral administration of 80 mg/kg AMX in gilthead seabream at 22–26 °C (Della Rocca et al. 2004). In the current study, the WDT in Nile tilapia at 25 and 30 °C were calculated as 6 and 4 days, respectively, and the time when the mean AMX concentration fell below the MRL were 5 and 3 days, respectively. Due to dissimilarity in the experimental conditions, a direct comparison among these studies cannot be made. However, it is reasonable to assume that during the tissue depletion phase, Nile tilapia eliminate AMX at a slower rate than Japanese eel, snubnose pompano, and gilthead seabream. More importantly, the 5-day WDT of AMX established by Taiwan’s Council of Agriculture (Executive Yuan) (COA 2023) following the standard dosing regimen (40 mg/kg/day for 5 days) may not be appropriate for Nile tilapia cultured at 25 °C or lower temperature.

## 5. Conclusion

The work is one of few studies that investigate the complete PK, optimal dosage, and WDT of AMX in fish. In addition, the effect of water temperature on PK and tissue residue of AMX was also assessed for the first time in any fish species. Our results demonstrated that water temperature exerts a substantial impact on the PK, optimal dosage, and WDT of AMX in Nile tilapia. An increase of 5 °C in temperature led to a two-fold acceleration in drug elimination rate and a more than three-fold increase in the optimal dosage. At the same time, the WDT following the standard dosing regimen (40 mg/kg/day for 5 days) was shortened by 2 days. The findings highlight the importance of water temperature which should be carefully taken into consideration when implementing drug treatments in real-life scenarios. Ignoring this factor could compromise treatment efficacy and consumer safety.

## Data Availability

The data that support the findings of this study are available from the corresponding author upon reasonable request.
